# Technology Courage: Implications for Faculty Development

**DOI:** 10.15694/mep.2018.0000144.1

**Published:** 2018-07-18

**Authors:** Virginia Niebuhr, Bruce Niebuhr, Anne Rudnicki, Mary Jo Urbani

**Affiliations:** 1 The University of Texas Medical Branch

**Keywords:** Technology courage, faculty development

## Abstract

This article was migrated. The article was marked as recommended.

**Purpose:** From a decade of technology-focused faculty development, the authors recognized that academic physicians adopt educational technology at varying rates and with variable confidence. This work is an exploration of the phenomenon of technology courage and how the concept can inform faculty development.

**Method:** Qualitative methods of interpretative phenomenological analysis (IPA) were used. Faculty interviews were transcribed using Google Docs voice typing. Data were analyzed, themes developed, and supportive narratives were identified using IPA methodology.

**Results:** Two themes emerged. The theme of Willingness includes willingness to try, explore, or risk learning a new technology; and willingness to persist in the face of fear or anxiety. The theme of Benefit Evaluation relates to motivators for technology courage, i.e., assessing benefit to self and learners before learning and using a new technology.

**Conclusions:** From a theme analysis, a definition of technology courage has emerged: willingness to try and to persist when using a new technology because of perceived benefit to self and/or others. The authors discuss how further research of the construct might be guided by theoretical frameworks of grit, self-efficacy, teacher identity, and generational learning differences. Recommendations are offered on how the construct of technology courage can be valuable for technology-focused faculty development

## Introduction

While conducting technology-focused faculty development over the last decade, we recognized that academic physicians adopt educational technology with varying degrees of ease. We have repeatedly been impressed with faculty’s willingness to venture and their perseverance in the face of difficulty while learning and using technology for educational activities.

One of our team, known for being very techy and an early adopter as defined by Rogers (2010) repeatedly looked for ways technology could help learning in the clinic and the classroom. He challenged others to do the same. In tribute, a retired colleague praised him for “giving me *technology courage.*”

This term technology courage was new to us. We asked, “Is this a phenomenon that actually exists? Can someone have technology courage? If so, could this term be useful for our faculty development endeavors?”

A literature search yielded no studies on technology courage, but we did find a lecture-based essay titled “Technology and Courage” in which Sutherland wrote “.. to do technology requires courage” ( Sutherland, 1996, p 6). He discussed several facets of courage in academia and business and made suggestions on how to gain more courage, especially when conducting academic research in the field of computer science. Citing Sutherland, Waldo (2006) asserted that what was lacking in the computer-system design industry is a lack of courage to take the kinds of risks inherent in system design. Other than these essays, we found no research and no other mention of technology courage, especially as a construct related to educational technology or faculty development. Although we believed technology courage exists, we had no clear definition of the term. We decided to ask recipients of our faculty development efforts to help define the term.

When a phenomenon needs exploration, phenomenological qualitative research methods are appropriate. The specific method of Interpretive Phenomenological Analysis (IPA)is appropriate when the phenomenon is examined through individuals’ experiences ( Pietkiewicz & Smith, 2014). The IPA method involves collection and analysis of participants’ insights about the phenomenon. There is no test of a predetermined hypothesis; but rather, the aim is to investigate how individuals make sense of their experiences. IPA has been widely used in the field of health psychology (e.g., investigating phenomena such as social support for people in pain, views of medical technologies by people with genetic conditions, and reactions to the death of a partner) ( Brocki & Wearden, 2006, Smith & Osborn, 2014).

The goal of our research was to explore the phenomenon of technology courage through investigation of the experiences of a sample of health professions educators. As we had identified a phenomenon yet had no hypotheses to test, the methods of IPA were determined to be most appropriate. We sought to identify definitions of and experiences with technology courage. We began with an inquiry - how do our participants define technology courage from their experiences? We then searched for evidence of constructs that make up the phenomenon of technology courage. Finally, we identified conceptual frameworks which might support further investigation of technology courage - frameworks of grit, self-efficacy, teacher identify, and generational learning differences. We believe our findings and discussion about technology courage can be useful for technology-focused faculty development for health professions educators.

## Methods

We used a phenomenological qualitative research methodology, specifically, Interpretive Phenomenological Analysis (IPA). As is appropriate to IPA, we conducted purposive sampling rather than random sampling ( Hanson, 2011). Grounding the investigation on our own experiences, we chose a sample of academic pediatric faculty with whom we had worked in our technology-focused faculty development program. True to IPA methods, data were collected from transcribed interviews, and analyzed through a process of reading and bracketing, and theme development. This project received ethical approval from the University of Texas Medical Branch Institutional Review Board (protocol reference number #16-0063).

**Participants.** Potential participants were full-time teaching faculty who had engaged in technology-focused faculty development in the Department of Pediatrics. Participants were recruited via email. Of the 24 potential participants contacted, 15 consented to be interviewed (63%) and the others either overtly declined or declined by nonresponse.

**Procedures.** An eight-item structured interview was developed and then piloted with a faculty member outside the sample. After the pilot interview, minor modifications were made in the questions. The interview questions are shown in Table 1. Three interviewers (AR, MJU, BN) were assigned to conduct three to five interviews each. Interviews were scheduled over a period of six weeks. Interviews were strictly structured, with no additional questions asked.

**Table 1.** Interview Questions

1	We are going to give you a two-word phrase...tell us what this phrase means to you: “technology courage”.
2	Can you describe a situation where you used technology as an educator and felt successful and why?
3	How about a situation where you used technology as an educator and did not feel successful or wished for a different experience?
4	When you get stuck using technology, what is your general strategy for getting unstuck? Please elaborate on your answer.
5	What technologies do you consider critical to your work as an educator?
6	Have you ever considered using an instructional technology and decided not to because of the learning curve? Please elaborate.
7	If you were to set a technology-related goal for yourself as an educator, to accomplish in the next 12 months, what might it be?
8	And a repeat question:We are going to give you a two-word phrase...tell us what this phrase means to you: “technology courage”.

Interviews were automatically transcribed using the voice-typing feature of Google Docs. This method eliminates the time-consuming and expensive need for human transcription, but does carry risks of internet interruption and voice misrecognition ( Rudnicki et al., 2017). Therefore, for backup, interviews were also audio-recorded using a digital audio recorder. Audio recordings were accessed to fill in missing text or correct any clearly incorrect transcription. Transcriptions and audio-recordings were stored on a password-protected server and deleted from the Google drive. After the interview, each participant was invited by email to complete an anonymous nine-item online demographic survey.

**Data Analysis.** Interview data were analyzed by the research team (i.e., the 4 authors) using the qualitative methodology of interpretative phenomenological analysis (IPA) ( Pietkiewicz & Smith, 2014).

**Step 1: Bracketing**. Individually, each researcher read an assigned subset of transcripts several times, also listened to audio recordings, and made exploratory notes. The primary author bracketed (i.e., read and made notes) all 15 transcripts.

**Step 2: Emergent themes.** Individually, each researcher developed initial themes from exploratory comments in their notes.

**Step 3: Clustering themes.** The researchers met together for a total of eight hours over four sessions to further analyze the data. They shared their individually determined initial emergent themes, merged similar themes, and discussed perceived differences. When support for a theme was unclear, researchers returned to the transcripts for substantiation and clarification. By full consensus, clustered themes were developed.

**Step 4: Graphic Organizer.** Using the clustered themes, a graphic organizer was developed for visual representation of the theme analysis.

**Step 5: Narrative account.** Each researcher reviewed their assigned transcripts for representative quotes to support the themes.

## Results/Analysis

**Demographics.** Participants were 15 pediatric faculty, eight were women and seven were men. Of the 15, 12 completed the online survey of demographics (80%). The age distribution was mostly even, with three in the 31-40 range, three in the 51-60 range, four in the 61-70 range, and two were over 70. The length of time on faculty was more skewed, with two new (2-5 years), two mid-range (6-10 years) and eight senior faculty (over 20 years).

**Theme development.** From the qualitative data analysis, two themes emerged: Willingness and Benefit Evaluation. See Table 2. In the following section, we illustrate the themes with direct quotes from interview data and interpretive description.

**Table 2.** Emergent Themes: Willingness and Evaluation of Benefits

Technology Courage			
Willingness		Evaluation of Benefit	
To try, explore, risk	Topersist	Benefit to others	Benefit to Self

**Willingness.** In responseto the question “what does the phrase ‘technology courage’ mean to you?” Faculty overwhelmingly referred to “willingness.” Responses clustered into two types of willingness:

**a. Willingness to Try.** Faculty responses were on a continuum of willingness to take some kind of action with technology-from solely trying technology, to exploring it, and finally to taking a risk with technology. Their definitions included: “Your ambition to learn new things having to do with technology in your day-to-day activities, and it probably has to do with forging new techniques, new ideas in relation to technology.” “A willingness to try new types of technology, new apps or programs..” “The ability or the wherewithal or the willingness to take risk in using technology.”

**b. Willingness to Persist, in the face of fear or anxiety.** Some faculty defined technology courage as a willingness to overcome fear, threats, challenges when using technology, including the willingness to stick with it. “I would infer that you would have the courage to use it..because it can be frightening sometimes.” “The courage to overcome the threats you may feel from new technology.” “A willingness to stick with it until you learn it.” “Technology courage is not being afraid to push limits to incorporating technology-related methods to your teaching endeavors..Step out of the comfort zone to bring those methods into your educational activities.”

Embedded in the theme of Willingness are references to strategies used to overcome threats, challenges and limits. What do faculty do when they are stumped? Many faculty told us they start by using a Google or YouTube search to get instructions for using a specific technology. “I Google it and see if I can find either a YouTube video or just maybe a quick blog entry or something where people have worked through the similar issue so I don’t give up..”

If such search strategies are not successful, faculty might seek help from an expert or someone who they feel knows more than they do about technology, including family members. “I go to an expert who I know may have been be more familiar with the technology.” “Find somebody who knows how to use it.” “If I really don’t know how to do it and Google’s not helping, and I think I know a person who would know that information, then I’ll email them.”

Rather than seeking out a sole expert, some faculty turn to their learners to “group source” the solution. “If I don’t know it, there [are] a couple other residents or faculty there .. we kind of group source it. Someone can kind of figure out what’s supposed to be done if it’s something really complex that I have no idea how to do.”

A few mentioned making sure they have a backup plan when using educational technology. “Last week I was preparing for lecture. I had the lecture all ready to go with audience response and the software is no longer downloadable. [then] I remembered that there are other methods that I have been instructed by experts. So, it’s good to have .. another system that I could go to in case things like this happen..”

Some faculty choose to free themselves to explore and “play” with the technology in order to get unstuck. “I have a series of things that I go through and it usually starts with play[ing] with the technology..even if you don’t solve the problem you started to solve, you learn more about how the technology works.” Sometimes faculty choose not to reach out to impersonal institutional support systems like a Help Desk because “it takes too long to get someone who knows what they’re doing.”

**Benefit Evaluation.** The theme of Benefit Evaluation describes what motivates faculty to use technology for teaching. When choosing whether to try a new technology for teaching, faculty evaluate the benefit to themselves and/or to others. We found benefit evaluation to be an iterative process for faculty. That is, faculty usually evaluate the benefit of using a technology before they have a willingness to try and then reevaluate the benefit at different points in the process.

Some faculty weighed the benefits both to themselves and to others when deciding if they would utilize technology in their teaching. “I know I have limits .. when it comes to application of technology, but I enjoy learning about it, and I know it makes education more interactive, or at least it can make it more interactive; and for this reason, I’ve tried to employ it [technology] whenever possible.” “I can teach with a piece of chalk and a chalkboard. I don’t need technology necessarily. If it’s a barrier then I’m just not going to use it.” “Being open and willing to sit down and learn something new that could be helpful not only to yourself but maybe to your peers and to your pupils.”

The benefit analysis theme emerged frequently when faculty spoke about specific technologies. For example, it was clear that several faculty had analyzed the benefit to others when choosing to integrate audience response systems (ARS) into their teaching. “When I first became aware of ARS, I immediately incorporated that approach and students really liked it because it keeps them engaged. It also tests how they’re receiving the material right at the moment.” “I think it [ARS] keeps people from falling asleep at 8:30 in the morning.” “I needed something [ARS] that was going to be very user-friendly especially for faculty that are going to be coming in on a monthly basis; and using it, then they would have to be able to navigate through it very quickly and easily without having much time training in it.”

The benefit analysis theme also showed when faculty spoke about the challenges of learners not being in the same place at the same time. “Using the Voice Thread application to facilitate a session with fourth year students who can’t be at every session.”

When exploring whether to learn a new technology, one faculty member weighed the learning curve and time required, and he found it was more productive and a better use of time to see patients, rather than learn a new way of making presentations interactive. “I can spend hours and hours; even if I master it, I still have to spend a lot of time creating it. It is always changing and I could spend more time seeing more patients.”

The following quote illustrates a faculty member’s evaluation of the benefit of using technology in her teaching. She analyzed how and why she will use multimedia effectively such that it does not overwhelm the learning process. “In a teaching session I give to the first-year interns.. I feel like I’ve got the balance of technology for smaller groups working well.. I’m using the technology but it’s not overwhelming the teaching session.. it is a very alive thing in that technology is enhancing rather than running the session..it feels like the technology is working for you and not you’re fighting it.”

## Discussion

This study was a phenomenological exploration of the construct of *technology courage.* Faculty provided us definitions of the term and described their experiences related to technology courage. From the data analysis, we conclude that faculty’s perceptions and experiences of technology courage clustered into two component constructs: Willingness and Benefit Evaluation.

Using the Hadi & Closs (2016) guidelines for trustworthiness of data, we believe our data reflects credibility, dependability, confirmability, and transferability. Credibility of our data and data analysis is high given the following elements: (a) the combination of our prolonged engagement in the realm of technology-focused faculty development and yet our open-mindedness for exploring a phenomenon new to us,(b) triangulation through the use of three interviewers and four data analyzers, (c) a consensus process for theme development, (d) analysis not made hastily, but occurring after many hours over several sessions, and (e) member checking/peer debriefing.

Dependability of our data was increased with the innovative use of Google Docs voice to text transcription. Confirmability of the results (i.e., would another researcher verify the results when presented with the same data) is high given that the themes emerged directly, and only, from the interview texts and we identified text narratives to support each theme description. Furthermore, confirmability is insured with the existence of both audio data and text data records. Finally, we believe transferability is high, at least among academic health center faculty. There is no reason to believe the experiences of technology courage are unique to pediatric faculty. Furthermore, other research groups who might want to explore the phenomenon with a different faculty group could easily duplicate our methods.

Faculty’s definitions of technology courage overwhelming included the construct of Willingness, including willingness to try, explore, risk, and persist. The construct of Willingness has previously arisen in work on teachers’ professional development, with evidence that for teachers to seek out the best teaching strategies, both willingness and a supportive environment are needed ( Wilson & Berne, 1999). Our faculty also spoke about supportive communities - needing others to bolster their courage and support their willingness to try.

Faculty’s definitions of technology courage also incorporated the theme of Benefit Evaluation, used to explain their motivations. Benefit Evaluation involves evaluating benefit both to self and to others. When using a new technology, Faculty evaluate the benefit for their own productivity, the effect of their effort on satisfaction with their roles as teachers, and the benefit for their learner’s satisfaction and success.

From our participants’ contributions, we have arrived at the following definition:Technology Courage is the willingness to try and to persist when using a new technology because of perceived benefit to self and/or others.

Of importance to us is how the construct of technology courage can be valuable for the work of faculty development. One participant stated, “I’m not sure that [technology courage] is something that you can learn.. you can learn how to do things, but your attitude about it may be kind of innate.” Maybe technology courage *per se* cannot be specifically taught or learned; but more important may be our recognition that technology courage affects learning and teacher identity. Faculty developers might be better coaches if they could identify how fearful vs. courageous a faculty member is about learning or applying a new technology. Our goal as faculty developers could be to help faculty be a little braver, a little more willing to try, and to help them move along in little steps.

Often faculty claim “I am technophobic” to explain their insecurities. Perhaps this negative tone could be turned more positive if faculty themselves and faculty developers began to think instead about technology courage and were aware of the constructs of willingness and benefit evaluation. Faculty developers could help faculty discover their personal levels of technology courage by helping them conduct a benefit evaluation and evaluate their willingness to try, thus turning ‘technophobic’ into ‘I’m anxious but willing to try because it’s important.’

Still unknown, yet certainly researchable, are what variables affect how technology courage develops and can be sustained. Future research might be guided by four conceptual frameworks - grit, self-efficacy, teacher identity, and generational learning differences.

We speculate that technology courage is related to the concept of grit, defined by Duckworth as perseverance and measurable with a grit scale ( Duckworth, Peterson, Mathews & Kelly, 2007, Duckworth, 2016). After re-examining our faculty interviews, we found evidence of grit from several of our faculty. One respondent specifically showed determined persistence to succeed when video-recording in residents’ clinics even in the face of multiple obstacles. She reported, “..we took a huge amount of creative approaches to get that set up..a really dogged sense of purpose and we got it done.”

We also speculate that technology courage is related to self-efficacy (i.e., Bandura’ concept of belief in one’s own ability to successfully perform a task; self-confidence) ( Bandura, 1977, 1994). A person with high self-efficacy believes s/he can perform a task successfully, views the task as attainable and will spend more effort gaining knowledge to complete the task. A person with low self-efficacy may avoid the task. Researchers may want to study the relationships between self-efficacy and one’s technology courage.

Maggio, Daley, Pratt & Torre (2018) have recently proposed that the online teaching/learning environment presents challenges to one’s teaching identity, what they refer to as “teaching identify dissonance.” Technology courage may buffer against this dissonance. The relationship of health professions teacher identity and technology courage is an area ripe for further investigation.

Is technology courage related to age? Our analysis revealed no obvious differences in technology courage across our wide range of ages. Although generational differences in learning with technology have been widely studied ( Lai & Hong, 2015, Waycott et al., 2010, Kirschner & De Bruyckere, 2017), debates continue on whether one’s generation is truly predictive of attitudes towards learning. Maybe it is not one’s generation that makes the difference, but instead one’s technology courage.

### Limitations

In retrospect, we could have asked our participants to self-asses their personal technology courage, with either a quantitative rating or a qualitative description. It may be awareness of one’s personal technology courage contributes to the success of technology-focused faculty development initiatives.

It might be questioned if this work is representative of all academic faculty, or skewed to pediatric faculty who might have differing amounts of willingness or make different benefit evaluation than other health professions educators. We can only speculate that our faculty are not that much different from educators in other health disciplines.

## Conclusion

Technology courage is a real phenomenon, recognized by faculty when learning and using technologies for education. Faculty show technology courage when they are willing to try a new educational technology application or are willing to risk a misstep with technology. They show technology courage when they persist after something goes wrong or when they face a steeper than expected learning curve. Evaluating the benefits of a technology application for self and learners is a requisite step for technology courage; and the trying, persisting, and evaluating benefit intertwine in an iterative process.

The phenomenon of technology courage is valuable for faculty development. We believe that if faculty are helped to overcome their fears and to make benefit evaluations, they can become more courageous. Awareness of the phenomenon of technology courage might help faculty developers provide more coaching, build in peer mentoring, use more patience, and encourage technology risk-taking.

## Take Home Messages

For faculty developers:

1. Help your participants consider their own technology courage, specifically their perceived willingness to try when a task gets very hard.
2. Walk your participants through a benefit evaluation to help them determine their motivation for using a specific technology.
3. Build a supportive environment for your faculty development activity or program. This might take the form of helpers walking around the room during a hands-on workshop, offers of ready access of assistance when working independently, or for developers to go to where the faculty are when they are struggling, face-to-face or remotely.

## Notes On Contributors

Dr. Virginia Niebuhr is a Distinguished Teaching Professor, medical educator, and pediatric psychologist, in the Department of Pediatrics at The University of Texas Medical Branch (UTMB), Galveston, TX. She has a national reputation for technology-focused faculty development.

Dr. Bruce Niebuhr is a retired Distinguished Teaching Professor and adjunct Professor of Pediatrics in the Department of Pediatrics, UTMB, Galveston, TX. His medical education career has been focused on research skills teaching and technology-focused faculty development.

Dr. Anne Rudnicki is former Assistant Professor and Distinguished Teaching Professor in the Department of Pediatrics, and former Senior Medical Educator in the Office of Educational Development at UTMB, Galveston, TX. She is currently working in the field of public school education.

Ms. Mary Jo Urbani is an instructional designer and systems analyst in the Department of Pediatrics at UTMB, Galveston, TX. She has been an integral part of the research team, assisting with interviews, qualitative analysis and manuscript writing.
