# Supplementary material for: Technology Courage: Implications for Faculty Development
Source: MedEdPublish (2016). 2018 Jul 18;7:144. [Version 1] doi: 10.15694/mep.2018.0000144.1 (PMC10701816; doi:10.15694/mep.2018.0000144.1)
Supplement: Peer review history

# Reviewer report: Reviewer response for version 1

**DOI:** 10.21956/mep.19615.r29163

**Published:** 2019-01-09

**Authors:** Mildred López

**Affiliations:** 1 Tecnologico de Monterrey, School of Medicine and Health Sciences

This review has been migrated. The reviewer awarded 4 stars out of 5 The authors present interesting findings regarding how faculty members adopt a technology. By being involved in the faculty development office, one learns that some teachers have that spark of explorers, and some are more reluctant to innovate. It was especially that curiosity that set me up to analyze medical educator’s professional identity, and it’s when I started my Ph.D. in Education. It is important to highlight the contribution of this paper, as it is a qualitative study that was performed following a structured methodology. Table 2 proposes a clear approach to define the term Technology Courage. I think the term could be extended to include the term innovation, rather than technology. When I was reading the first paragraphs I thought about several factors affecting the study such as age and medical specialty, and those were explained in the limitations section. I still think that the discussion section could improve by incorporating the part of “implications for faculty development”. Trying to explain, according to the authors' experience, what can faculty developers do to ease this process.

Reviewer Expertise:

NA

No decision status is available

# Reviewer report: Reviewer response for version 1

**DOI:** 10.21956/mep.19615.r29161

**Published:** 2018-07-20

**Authors:** Sateesh Babu Arja

**Affiliations:** 1 Avalon University School of Medicine

This review has been migrated. The reviewer awarded 4 stars out of 5 This article is a well written paper. I enjoyed reading this paper. Nowadays simulation and technology are inseparable from education. Technology and simulation became very crucial in the medical education and training. Not only the technology, generally academic community and medical faculty resist the change in general. It always depends on the change process. It is how well the change is integrated. They are ready to learn if the change process is well informed and provided the right information and make them as part of the change process. Faculty development activities are crucial when changes are implemented and new technologies are introduced. According to the diffusion theory by Rogers, E. M. (2003) Diffusion of Innovations (5th ed.) New York, NY, Free Press, in the business market, there are 2.5% innovators, 13.5% early adopters, 34% early majority, 34 late majority, and 16% laggards. I don't see any reason not to be applied this in the health professions education too. This article is useful for faculty members and academic administrators.

Reviewer Expertise:

NA

No decision status is available

# Reviewer report: Reviewer response for version 1

**DOI:** 10.21956/mep.19615.r29160

**Published:** 2018-07-18

**Authors:** P Ravi Shankar

**Affiliations:** 1 American International Medical University

This review has been migrated. The reviewer awarded 4 stars out of 5 I enjoyed reading this paper. New technology is being introduced at a dramatic pace. Technology, the internet and online resources are now an indispensable component of learning. When I was younger I was better able to adopt new technology. Now I am older and do struggle with some technologies. Teachers and professors would like to be in control and masters of the situation. Modern teaching-learning methods democratize the classroom and students play an increasingly important role in their own learning. Some teachers still find this challenging. I have been involved in faculty development programs in Nepal, India and the Caribbean and find some faculties are ready adopters of new technology while others struggle. Students are often good with new technology and if in doubt in the classroom I find getting help from students may be often helpful. The methods section is comprehensive and the authors introduced me to voice typing which I plan to try out soon. I would be interested in more details on how the authors used this function to transcribe their interviews. All medical educators will find this paper to be of interest.

Reviewer Expertise:

NA

No decision status is available

# Reviewer report: Reviewer response for version 1

**DOI:** 10.21956/mep.19615.r29162

**Published:** 2018-07-18

**Authors:** Trudie Roberts

**Affiliations:** 1 Leeds Institute of Medical Education

This review has been migrated. The reviewer awarded 3 stars out of 5 When I first read this paper I wasn’t keen on the term Technological courage and thought that curiosity or adventurousness was better but I looked up the definition of courage and came across this: the ability to do something that frightens one; bravery. So I think the authors are correct in using this term. I enjoyed reading this paper and agree with the authors that Faculty Development and support to use new technology is essential. It would have been useful to know whether time constraints were also a barrier to adopting new technology and whether staff considered weighed the ‘pain’ of learning something new against the potential gain and whether the fear of failure in front of students played a part

Reviewer Expertise:

NA

No decision status is available
